# Determination of Polybrominated Diphenyl Ethers in Water Samples Using Effervescent-Assisted Dispersive Liquid-Liquid Icroextraction with Solidification of the Aqueous Phase

**DOI:** 10.3390/molecules26051376

**Published:** 2021-03-04

**Authors:** Yue Wang, Qicai Zhang, Shanshan Chen, Lin Cheng, Xu Jing, Xianli Wang, Shuhui Guan, Weiguo Song, Qinxiong Rao

**Affiliations:** 1 College of Food Sciences, Shanghai Ocean University, Shanghai 201306, China; wywy1005@126.com; 2 Institute for Agro-food Standards and Testing Technology, Shanghai Academy of Agricultural Science, Shanghai 201403, China; qicaizhang@126.com (Q.Z.); cssm100@163.com (S.C.); chenglin_8813@126.com (L.C.); wangxianli@saas.sh.cn (X.W.); shuhuiguan@163.com (S.G.); 3 College of Food Science and Engineering, Shanxi Agricultural University, Taigu, Jinzhong 030801, China; x.jing@vip.163.com

**Keywords:** polybrominated diphenyl ethers, effervescent-assisted dispersion, liquid-liquid microextraction, solidification of the aqueous phase, water samples

## Abstract

An effective and sensitive method is necessary for the determination of polybrominated diphenyl ethers (PBDEs) pollutants in water. In this study, effervescent-assisted dispersive liquid-liquid microextraction with solidification of the aqueous phase (EA-DLLME-SAP), followed by Gas Chromatography-Tandem Mass Spectrometry (GC-MS-MS) quantitative analysis, was established for the preconcentration and determination of PBDEs in real environmental water samples. 1,1,2,2-Tetrachloroethane was used as the extractant and directly dispersed into the water phase of the aqueous samples with the aid of a large number of carbon dioxide bubbles generated via the acid-base reaction of acetic acid and sodium bicarbonate, which did not require the use of a dispersant during the extraction process. The key factors affecting the extraction recovery were optimized, and an internal standard was used for quantitative analysis, which gave good linearity ranges of 1–100 ng·L^−1^ (BDEs 28, 47, 99, and 100), 2–200 ng·L^−1^ (BDEs 153, 154, and 183) and 5–500 ng·L^−1^ (BDE 209) with limits of quantification in the range of 1.0–5.0 ng·L^−1^. The accuracy was verified with relative standard deviations < 8.5% observed in tap, lake, river and reservoir water samples with relative recoveries ranging from 67.2 to 102.6%. The presented method contributes to the determination of PBDEs in environmental water samples.

## 1. Introduction

Polybrominated diphenyl ethers (PBDEs), which have 209 theoretical congeners, are widely used as flame retardants in furnishings, wood, paper and textiles, especially in electronic products, due to their low price and excellent fire resistance [1]. Among them, common commercial mixtures of penta-BDE, octa-BDE and deca-BDE were listed in the Stockholm Convention in 2004, 2004 and 2019, respectively [2]. PBDEs are persistent organic pollutants with toxic, persistence and bioaccumulation properties as well as long-range transport characteristics [3]. According to toxicological experiments, PBDEs can cause negative effects on the endocrine, nervous and reproductive systems of organisms in the food chain [4,5]. They can be easily combined with suspended particles and transferred to wet or dry fluxes with the movement of water or the atmosphere [6]. Studies showed that BDE 28, 47, 99, 100, 153, 154, 183 and 209 have been detected in various environmental media, such as water, dust, sediment, sludge, effluent and organisms [7,8,9,10]. Water is a key environmental hub causing the wide spread of PBDEs with eight PBDEs in a concentration range of 0.1–254 ng·L^−1^ [9].Therefore, it is particularly important to develop efficient and reliable methods for the detection of trace amounts of PBDEs in water [11].

Several methods have been reported for the determination of trace amounts of PBDEs in water samples based on Gas Chromatography-Tandem Mass Spectrometry (GC-MS-MS) or Gas Chromatography-Mass Spectrometry (GC-MS), and liquid-liquid extraction (LLE) or solid-phase extraction (SPE) have been applied for the pretreatment of water samples [12,13,14,15,16]. In these studies, usually 1 L of water is collected for each sample and 120 mL of solvent is consumed to extract PBDEs from the sample, and a large amount of time is spent carrying out the procedure. As an alternative solution to save time and solvent, various types of extraction techniques have emerged, such as liquid phase microextraction (LPME), solid phase microextraction (SPME) and stir bar adsorption extraction (SBSE), among which LPME techniques are the most widely used [17,18]. Dispersive liquid-liquid microextraction (DLLME), a ternary solvent system [19], is an innovation based on LPME, which exhibits high extraction efficiencies under the assistance of various auxiliary treatment steps, such as vortex, ultrasound, microwave and air [20,21,22,23]. However, these assistance technologies usually require the use of additional equipment or are labor-intensive. In order to overcome these shortcomings, effervescent-assisted dispersive liquid-liquid microextraction (EA-DLLME), a fast and efficient method [24], was developed to promote the extraction process using carbon dioxide bubbles generated from the reaction between acid and carbonate/bicarbonate [25,26]. Therefore, EA-DLLME may be a simple and quick technique used for the extraction of PBDEs in water.

To guarantee that the organic phase can be completely removed, dispersive liquid-liquid microextraction based on the solidification of floating organic drop (DLLME-SFO) technology has emerged in order to achieve efficient two-phase separation [27]. By using SFO technology, the extraction solvents can float on the surface of the water phase and be collected from the water phase [27,28]. However, only low-density organic solvents with a melting point close to room temperature can be employed in this method, and the scope of its application is limited [29,30]. Based on DLLME-SFO, dispersive liquid-liquid microextraction with solidification of the aqueous phase (DLLME-SAP) was invented by March and Cerdà in 2016 to expand the range of organic solvents and to be less restricted by the melting point [31]. The water phase can be completely solidified so that the organic phase is separated and transferred [32,33]. Thus, the combination of effervescent extraction, aqueous phase solidification and DLLME technologies enables the complete collection of the organic phase, thereby allowing the efficient and environmentally friendly analysis of PBDEs in aqueous samples.

Eight vital PBDEs (BDE 28, 47, 99, 100, 153, 154, 183 and 209) are not easy to be extracted simultaneously from environmental water samples because they contain different kinds of bromine functional groups and show distinct physical and chemical properties [34]. However, DLLME technology has been widely used in water, food, natural product and pharmaceutical and biomedical analyses [35,36]. In this study, a method combining effervescent-assisted dispersive liquid-liquid microextraction with the solidification of the aqueous phase (EA-DLLME-SAP) and GC-MS-MS analysis was developed to solve the problem of fast and effective dispersion of the extractant added to aqueous samples. Various factors possibly affecting the extraction efficiency were considered, and key parameters of the EA-DLLME-SAP method such as correlation coefficients and limit of quantification (LOQ) were validated following the applicability investigation in four different water samples. In addition, the performance of this method was compared with other technologies.

## 2. Results and Discussion

### 2.1. Optimization of EA-DLLME-SAP Parameters

#### 2.1.1. The Effect of Different Extraction Solvents

**Figure 1:**
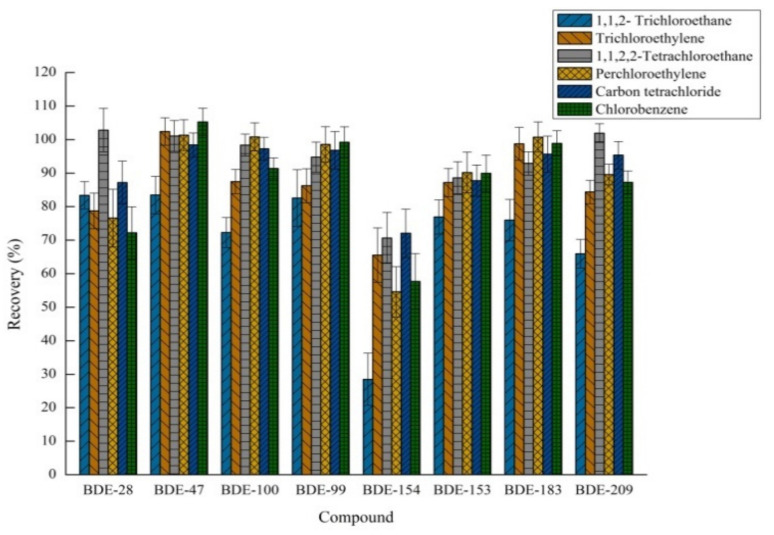
Effect of the type of extraction solvent on recovery (*n* = 3).

In order to achieve a high extraction efficiency in EA-DLLME-SAP when selecting the appropriate extraction solvent, it was necessary to optimize the process based on its low water solubility, strong extraction capacity and excellent GC-MS-MS behavior. Upon considering these three conditions, seven solvents including dichloromethane, 1,1,2-trichloroethane, trichloroethylene, 1,1,2,2-tetrachloroethane, chlorobenzene, perchloroethylene and carbon tetrachloride were verified as the extraction solvent. Other invariable factors were 80.0 μL of extractant, 85 μL of acetic acid and 75 mg of sodium bicarbonate. As it is difficult for dichloromethane to form suspended droplets and then a sedimented phase after centrifugation, the effects of the other six extractants were studied, as shown in Figure 1. 1,1,2-Trichloroethane had the lowest extraction recovery for eight compounds. When compared with the remaining four extractions, 1,1,2,2-tetrachloroethane displayed a significant extraction effect on BDE 28. Therefore, 1,1,2,2-tetrachloroethane was chosen as the extraction solvent for this system considering the overall extraction efficiency of the target compounds.

#### 2.1.2. Effects of Extraction Solvent Volume

**Figure 2:**
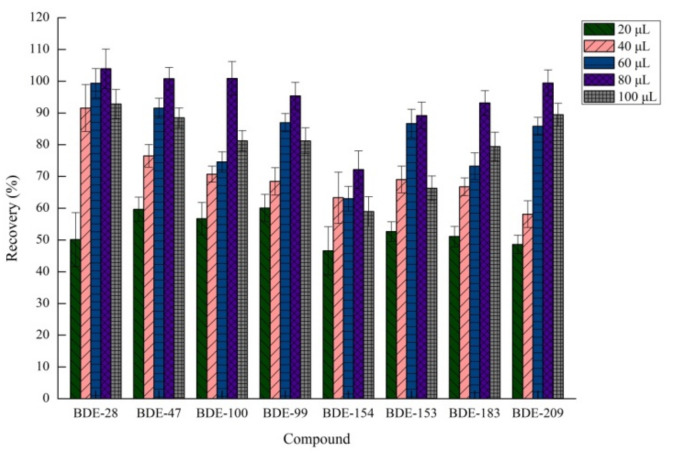
Effect of volume of extraction solvent on recovery (*n* = 3).

The extraction solvent volume is one of the possible variables in microextraction procedures. The compounds were tested by varying the volume of 1,1,2,2-tetrachloroethane in the range of 20–100 μL in 20 μL intervals. Other invariable factors were 85 μL of acetic acid and 75 mg of sodium bicarbonate. As shown in Figure 2, when the volume of the extraction solvent was 80 μL, the extraction efficiency was the best, and the PBDEs exhibited high distribution coefficients. When the volume of the extraction solvent was >80 μL, the recovery of the eight target compounds decreased. As a result, 80 μL of 1,1,2,2-tetrachloroethane was used in our further experiments.

#### 2.1.3. Effects of Sodium Bicarbonate

**Figure 3:**
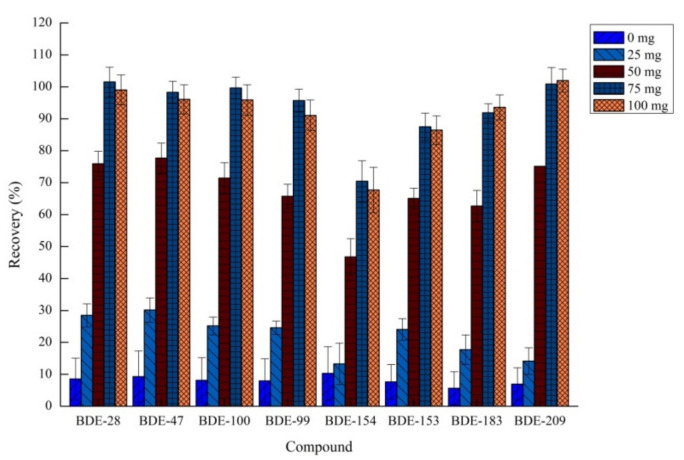
Effect of amount of sodium bicarbonate on recovery (*n* = 3).

Sodium bicarbonate has a vital role in the effervescent reaction. The amount of carbon dioxide produced increases upon the addition of sodium bicarbonate, which greatly promotes mass transfer, thereby improving the extraction efficiency [37,38,39]. The amount of sodium bicarbonate was investigated in the range of 0–100 mg to study the extraction efficacy. Other invariable factors were 80.0 μL of 1,1,2,2-tetrachloroethane and 85 μL of acetic acid. The results shown in Figure 3 indicated that the extraction ability improved as the amount of sodium bicarbonate increased, and the maximum recovery rate was achieved at 75 mg sodium bicarbonate addition. When the amount of sodium bicarbonate was >75 mg, the recovery did not change significantly. Hence, 75 mg of sodium bicarbonate was selected in our further experiments.

#### 2.1.4. Effects of Volume of Acetic Acid

**Figure 4:**
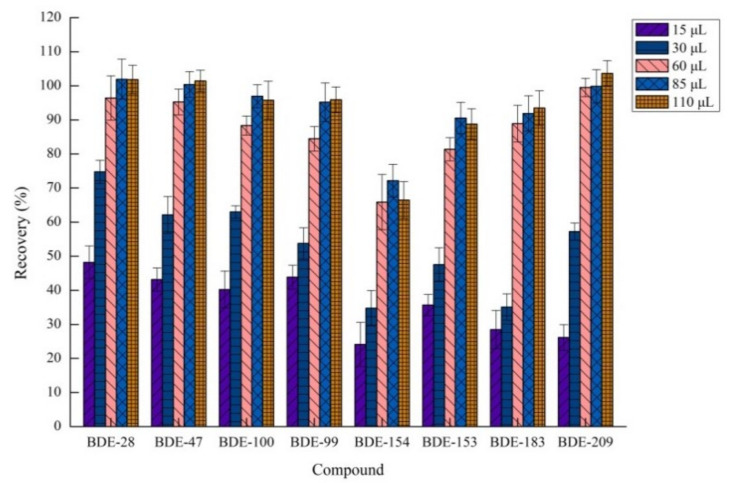
Effect of volume of acetic acid on recovery (*n* = 3).

The effervescent reaction between sodium bicarbonate and acetic acid determines the amount of CO_2_, which affects the extraction efficiency [40]. Therefore, the effect of the volume of acetic acid was studied in the range of 15–110 μL. Other invariable factors were 80.0 μL of 1,1,2,2-tetrachloroethane and 75 mg of sodium bicarbonate. The results shown in Figure 4 indicated that the maximum recovery was obtained using 85 μL of acetic acid because 75 mg of sodium bicarbonate exactly reacted with acetic acid to produce sufficient CO_2_ and the extraction effect was the best. A moderate volume of acetic acid (85 μL) was employed in the effervescence procedure.

#### 2.1.5. Effects of Sodium Chloride Amount

**Figure 5:**
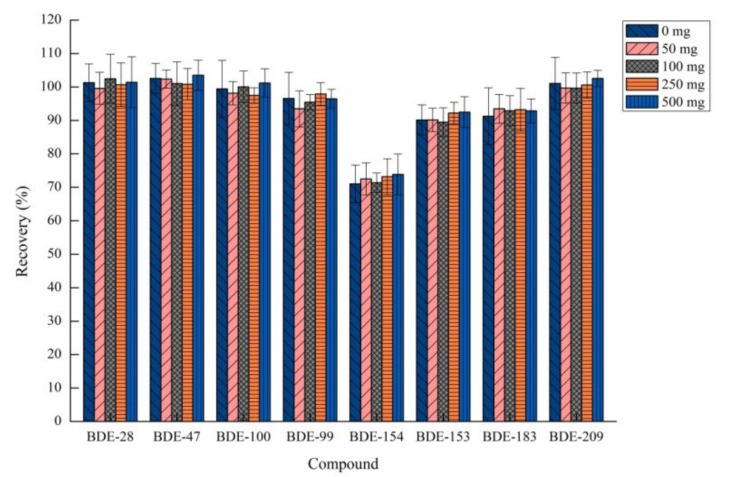
Effect of amount of sodium chloride on recovery (*n* = 3).

The effect of salt addition on extraction efficiency varies depending on the chemical nature of the extraction system [41]. The recycling ability of the ionic strength on the PBDE response was studied by adding different amounts of salt in the range of 0–500 mg. Meanwhile, all the other process conditions remained unchanged. The results in Figure 5 showed that increasing the salt content had no significant effect on the extraction efficiency of the target compounds studied in this experiment. Ultimately, no additional salt was found to be the optimal parameter in this EA-DLLME-SAP procedure.

### 2.2. Method Validation

Under optimal EA-DLLME-SAP conditions, the analytical characteristics including linearity, correlation coefficients, EF, precision and LOQ were evaluated with GC-MS-MS (Table 1). The enrichment factors were calculated according to the following equation:

(1) $$\mathrm{EF}=\frac{V_{\mathrm{sample}}}{V_{\mathrm{sed}}}\times \mathrm{ER}$$

where $V_{\mathrm{sample}}$ and $V_{\mathrm{sed}}$ are the volume of the sample solution and the volume of sedimented phase, respectively. The extraction recovery was calculated according to the equation:

(2) $$\mathrm{ER}\;=\frac{C_{1,1,2,2-\mathrm{tetrachloroethane}}\;\times {\;V}_{1,1,2,2-\mathrm{tetrachloroethane}}}{C_{\mathrm{sample}\;}\times {\;V}_{\mathrm{sample}}}\times 100\%,$$

where$\;C_{1,1,2,2-\mathrm{tetrachloroethane}}$,$\;C_{sample}$,$\;V_{1,1,2,2-\mathrm{tetrachloroethane}}$ and $V_{sample}$ are the concentration of PBDEs in the 1,1,2,2-tetrachloroethane phase, the initial concentration of the PBDEs in the sample solution, the volume of the 1,1,2,2-tetrachloroethane phase and the volume of the sample solution, respectively. The calibration curves were assessed by three replicate extractions at concentrations in the range of 1–100 ng·L^−1^ for BDEs 28, 47, 99 and 100; 2–200 ng·L^−1^ for BDEs 153, 154 and 183; and 5–500 ng·L^−1^ for BDE 209 with the correlation coefficient >0.9991. The LOQ, based on the lowest concentration point on the calibration curve according to methods in EU Regulation 709/2014 [42], ranged from 1.0 to 5.0 ng·L^−1^ for the PBDEs. The EF values were obtained in the range of 68.5–102.5 for the four different types of water samples studied. The precision of this study was verified using intra-day and inter-day (*n* = 6) experiments. The spiked concentration of BDEs 28, 47, 99 and 100, BDEs 153, 154 and 183 and BDE 209 in intra-day and inter-day RSD evaluation were 10 ng·L^−1^, 20 ng·L^−1^ and 50 ng·L^−1^, respectively. The inter-day RSD ranged between 1.8 and 8.2% and the intra-day RSD between 1.9 and 7.6%, implying that the proposed technique has the ability to accurately analyze the target compounds.

### 2.3. Application to Water Sample Analysis

In order to investigate the applicability of EA-DLLME-SAP in environmental aqueous samples, three concentrations of PBDEs in tap, lake, river and reservoir water were extracted and analyzed. As shown in Figure S1, no residual PBDEs (<LOQ) were observed in the blank control water samples. The recovery rate of water samples spiked with different concentrations of the PBDEs was in the range of 67.2–102.6% with the RSDs observed in the range of 1.9–8.5% (Table 2). As a result, real environmental water samples can be treated utilizing EA-DLLME-SAP and detected using GC-MS-MS.

### 2.4. Comparison of EA-DLLME-SAP with Other Extraction Techniques

The performance of EA-DLLME-SAP for the determination of PBDEs in water was compared with other published extraction technologies, comparing the volume of extraction solvent, dispersive solvent and sample, extraction time, linearity range, LOD and recovery (Table 3). It can be seen that the effervescent-assisted microextraction method did not require the addition of dispersants other than CH_3_COOH and NaHCO_3_ or the use of auxiliary equipment such as an ultrasonic cleaning machine or microwave oven [43,44,45]. At the same time, when compared with traditional solid-phase extraction [14] and liquid-liquid extraction [46] methods, EA-DLLME-SAP used only 80 μL of the extraction solvent to extract the target PBDEs from water, which exhibited high efficiency and used fewer solvents. In addition, the LOD of EA-DLLME-SAP-GC-MS-MS in this study was lower than UA-DLLME-GC-NCI-MS, SFOME-HPLC-VWD, DLLME-HPLC-VWD, SPE-DLLME-GC-ECD and EA-DLLME-SW-GC-MS-MS. The extraction time of the presented method was extremely short (<30 s), which greatly reduced the pretreatment time. Overall, it was confirmed that EA-DLLME-SAP is a relatively highly efficient, low-cost, sensitive method for detecting PBDE residues in water samples.

## 3. Materials and Methods

### 3.1. Chemicals

A standard mixture of eight PBDE congeners (BDE 28, 47, 99, 100, 153, 154, 183 and 209) in a mixed solution (BDEs 28, 47, 99 and 100 (0.500 mg·L^−^^1^); BDEs153, 154 and 183 (1.000 mg·L^−^^1;^); BDE 209 (2.500 mg·L^−^^1^)) and a surrogate standard MBDE-MXG (13C12-BDE 28, 47, 99, 100, 153, 154, 183 and 209) in a mixed solution (500 μg·L^−1^) were obtained from Accustandard (New Haven, CT, USA). Chromatographic grade hexane, dichloromethane and acetonitrile were purchased from Merck Company (Darmstadt, Germany). Acetic acid (17.4 mol·L^−1^), 1,1,2-trichloroethane, trichloroethylene, 1,1,2,2-tetrachloroethane, perchloroethylene, carbon tetrachloride, sodium chloride and sodium bicarbonate were purchased as analytical grade solvents/reagents from Sinopharm Chemical Reagent (Shanghai, China). Chlorobenzene was obtained from Macklin Biochemical Technology Co. (Shanghai, China).

The working standard solutions of each compound were re-dissolved in acetonitrile and stored at −20 °C in amber glass flasks. The standard solutions were diluted step by step with the appropriate dilution of the stock solutions in acetonitrile. All the stock solutions were maintained at 4 °C in the dark.

### 3.2. Instrument

PBDEs were determined on a GC-MS-MS instrument (TSQ9000, Thermo, Austin, TX, USA) in Advanced Electron Ionization (AEI) mode. The GC system was coupled to a DB-5HT capillary column (15 m × 0.250 mm I.D., 0.10 μm film thickness, Agilent, Shanghai, China). The injection was performed in splitless PTV mode in a sample volume of 2.0 μL and the septum purged at a constant flow rate of 5.0 mL·min^−1^. Helium was employed as the carrier gas at a constant flow rate of 1.5 mL·min^−1^ with both the MS transfer line and ion source temperature at 280 °C. The GC oven temperature was started at 100 °C (held for 2 min) and then the column heated to 340 °C at a heating rate of 30 °C·min^−1^ with the final temperature held for 3 min. The GC-MS-MS optimized parameters resulted in the retention times of BDE 28, 28-IS, 47, 47-IS, 99, 99-IS, 100, 100-IS, 153, 153-IS, 154, 154-IS, 183, 183-IS, 209 and 209-IS of 6.07, 6.07, 6.79, 6.79, 7.30, 7.30, 7.45, 7.45, 7.85, 7.85, 8.04, 8.04, 8.61, 8.61, 10.71 and 10.71 min, respectively.

### 3.3. EA-DLLME-SAP Procedure

**Figure 6:**
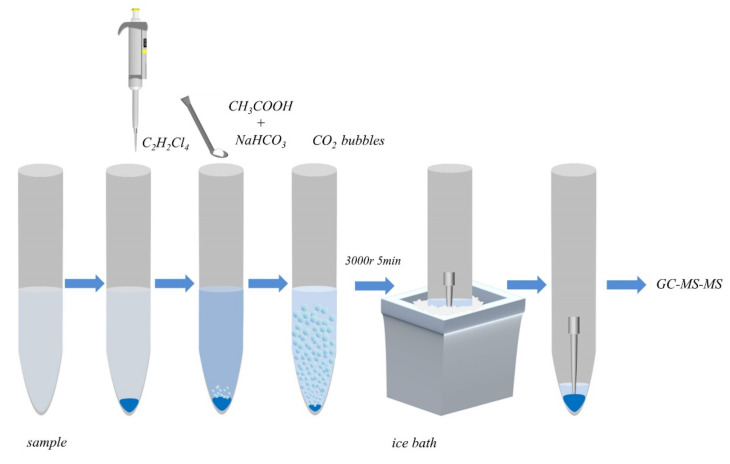
Schematic diagram of the EA-DLLME-SAP procedure.

A total of 5.0 mL of the aqueous sample solution was placed into a 10 mL glass centrifuge tube with a conical bottom. Then, under optimal parameter conditions, 80.0 μL of 1,1,2,2-tetrachloroethane (as an extraction solvent), 85 μL of acetic acid (17.4 mol·L^−1^) and 75 mg of sodium bicarbonate were added sequentially to the test tube. During effervescent-assisted extraction (<30 s), 1,1,2,2-tetrachloroethane homogeneous suspended droplets were produced and completely dispersed as a result of the generation of carbon dioxide (CO_2_). Finally, the cloudy solution was centrifuged at 3000 rpm for 5 min, discarding most of the water in the upper layer in the test tube to 3–5 mm higher than the organic phase. The pipette tip was inserted into the liquid, and the centrifuge tube was put into a −18 °C cooling bath and frozen for 10 min until the water phase was completely solidified, which ensured that the sedimented phase was completely taken out after placing in the cooling bath. The final collected 74 ± 2 µL organic phase was concentrated to dryness under a gentle nitrogen flow, and then was re-dissolved in a mixed solution of 10 μL of MBDE-MXG and 40 μL of hexane prior to GC analysis. Figure 6 shows the microextraction procedure used in the EA-DLLME-SAP method.

## 4. Conclusions

In the study, an efficient alternative microextraction method using effervescent-assisted dispersive liquid-liquid microextraction based on solidification of the aqueous phase (EA-DLLME-SAP) extraction and GC-MS-MS analysis was developed for the preconcentration and determination of PBDE residues in aqueous samples. Under optimal parameters, the effervescent reaction could generate a large amount of carbon dioxide to achieve good sensitivity, high efficiency, low solvent consumption and environmentally friendly auxiliary extraction. The developed method eliminated the use of dispersants and gave good linearity in the range of 1–100 ng·L^−1^ (BDEs 28, 47, 99 and 100), 2–200 ng·L^−1^ (BDEs 153, 154 and 183) and 5–500 ng·L^−1^ (BDE 209) with RSDs ranging from 1.9 to 8.5%. Therefore, EA-DLLME-SAP-GC-MS-MS with internal standard quantitative analysis has great potential in the field of trace multi-PBDE residue analysis in diverse environmental water samples.

## Figures and Tables

**Table 1:** Analytical Performance of EA-DLLME-SAP Coupled with GC-MS-MS Analysis.

Analyte	Sample	Linear Equation (ng·L^−1^)	R^2^	LOQ (ng·L^−1^)	Intra-Day RSD (%) (*n* = 6)	Inter-Day RSD (%) (*n* = 6)	EF
**BDE-28**	Tap water	y = (0.0321 ± 0.0011)x − 0.0014	0.9991	1.0	5.1	4.5	101.5
	Lake water	y = (0.0367 ± 0.0015)x − (0.0062 ± 0.0001)	0.9991	1.0	4.2	2.9	101.2
	River water	y = (0.0336 ± 0.0012)x − (0.0044 ± 0.0001)	0.9991	1.0	4.6	4.1	102.3
	Reservoir water	y = (0.0283 ± 0.0013)x − (0.0039 ± 0.0001)	0.9997	1.0	6.5	3.3	100.8
**BDE-47**	Tap water	y = (0.0193 ± 0.0007)x + 0.0019	0.9998	1.0	2.2	3.8	100.6
	Lake water	y = (0.0204 ± 0.0012)x − 0.0006	0.9999	1.0	3.8	4.2	102.5
	River water	y = (0.0193 ± 0.0009)x + 0.0009	0.9999	1.0	3.1	6.5	101.9
	Reservoir water	y = (0.0176 ± 0.0010)x + 0.0005	0.9999	1.0	5.2	5.1	101.7
**BDE-100**	Tap water	y = (0.0205 ± 0.0009)x + 0.0016	0.9999	1.0	4.9	5.6	98.6
	Lake water	y = (0.0214 ± 0.0014)x + 0.0007	0.9999	1.0	5.6	4.3	99.1
	River water	y = (0.0210 ± 0.0007)x + 0.0004	0.9999	1.0	4.6	6.9	98.4
	Reservoir water	y = (0.0192 ± 0.0009)x − 0.0007	0.9999	1.0	3.9	3.1	99.2
**BDE-99**	Tap water	y = (0.0182 ± 0.0008)x + 0.0009	0.9999	1.0	3.2	5.2	95.2
	Lake water	y = (0.0193 ± 0.0006)x + 0.0015	0.9998	1.0	2.5	4.9	94.6
	River water	y = (0.0190 ± 0.0008)x + 0.001	0.9999	1.0	3.8	3.2	95.7
	Reservoir water	y = (0.0170 ± 0.0009)x + 0.0006	0.9999	1.0	2.4	4.6	94.1
**BDE-154**	Tap water	y = (0.0147 ± 0.0011)x + (0.0015 ± 0.0001)	0.9999	2.0	6.1	5.9	70.6
	Lake water	y = (0.0177 ± 0.0008)x − (0.0033 ± 0.0001)	0.9999	2.0	5.8	7.6	68.5
	River water	y = (0.0160 ± 0.0007)x − 0.0008	0.9992	2.0	7.6	4.2	71.2
	Reservoir water	y = (0.0146 ± 0.0004)x + 0.0015	0.9999	2.0	2.3	3.4	70.5
**BDE-153**	Tap water	y = (0.0137 ± 0.0010)x + 0.0004	0.9997	2.0	3.4	2.8	88.6
	Lake water	y = (0.0150 ± 0.0006)x − 0.0001	0.9998	2.0	1.9	8.2	87.9
	River water	y = (0.0132 ± 0.0008)x + (0.0032 ± 0.0001)	0.9998	2.0	3.4	4.8	89.2
	Reservoir water	y = (0.0134 ± 0.0003)x − 0.0014	0.9999	2.0	2.8	6.7	88.1
**BDE-183**	Tap water	y = (0.0097 ± 0.0006)x − 0.0002	0.9999	2.0	2.3	2.3	91.8
	Lake water	y = (0.0101 ± 0.0004)x − 0.0022	0.9999	2.0	4.5	7.5	93.5
	River water	y = (0.0089 ± 0.0004)x − 0.0012	0.9999	2.0	6.6	3.4	92.6
	Reservoir water	y = (0.0090 ± 0.0005)x − 0.0010	0.9999	2.0	4.8	3.4	93.1
**BDE-** **209**	Tap water	y = (0.0045 ± 0.0001)x − 0.0004	0.9999	5.0	3.5	5.9	100.3
	Lake water	y = (0.0042 ± 0.0001)x + (0.0062 ± 0.0001)	0.9999	5.0	2.8	2.6	101.6
	River water	y = (0.0045 ± 0.0002)x − (0.0024 ± 0.0001)	0.9999	5.0	5.1	1.8	102.5
	Reservoir water	y = (0.0037 ± 0.0002)x + 0.0007	0.9999	5.0	3.8	3.2	101.4

**Table 2:** Analysis of PBDEs in Water Samples.

		Tap Water		Lake Water		River Water		Reservoir Water	
Analyte	Concentration (ng·L^−1^)	Recovery (%)	RSD (%)	Recovery (%)	RSD (%)	Recovery (%)	RSD (%)	Recovery (%)	RSD (%)
**BDE-28**	1	101.1	5.8	100.5	4.6	99.8	4.7	100.3	3.8
	10	99.8	3.6	102.1	6.5	100.4	4.6	102.6	5.1
	100	100.6	2.5	101.4	4.5	99.5	4.8	102.4	4.3
**BDE-47**	1	101.3	4.3	99.5	7.6	100.6	4.8	101.5	1.9
	10	100.1	6.7	99.1	4.8	99.8	7.1	100.7	5.6
	100	100.8	4.2	100.3	3.5	101.1	4.5	100.9	4.2
**BDE-100**	1	98.4	3.9	95.9	4.5	97.2	3.5	96.5	5.1
	10	96.5	4.5	98.7	4.1	96.8	5.4	99.2	2.1
	100	99.1	4.2	97.2	4.8	98.9	4.8	98.3	5.6
**BDE-99**	1	95.5	2.8	93.7	2.2	95.3	3.5	95.2	4.6
	10	97.2	3.1	94.2	6.2	93.1	4.3	94.5	3.8
	100	96.8	3.2	94.5	4.3	94.8	4.5	93.8	3.4
**BDE-154**	2	71.2	4.1	68.2	8.5	67.2	5.9	70.1	5.7
	20	69.4	7.6	70.3	7.8	72.1	6.8	68.5	6.4
	200	70.6	3.9	70.8	5.1	71.6	8.4	69.2	4.6
**BDE-153**	2	90.1	4.5	87.6	3.1	88.1	3.5	89.2	5.2
	20	87.7	3.6	88.9	4.8	90.2	4.5	87.6	4.5
	200	88.6	3.4	88.6	3.6	89.4	4.2	89.5	8.5
**BDE-183**	2	91.9	5.4	91.3	6.4	93.5	2.8	91.8	7.8
	20	92.9	3.5	94.3	4.8	91.6	6.1	93.2	5.6
	200	93.2	6.5	92.6	5.3	93.1	4.6	92.8	4.5
**BDE-209**	5	99.5	4.9	98.6	5.2	98.4	4.3	99.2	5.8
	50	101.8	3.1	101.3	3.1	100.4	3.6	100.6	3.6
	500	100.6	5.6	99.8	3.2	99.5	2.4	101.4	4.3

**Table 3:** Comparison of the EA-DLLME-SAP method with other reported approaches for PBDEs.

Methods	Dispersive Solvent	Sample Volume (mL)	Extraction Time (min)	Centrifuge Time (min)	Solidification Time (min)	Injection Time (min)	Linearity Range (ng·L^−1^)	Recovery(%)	Reference
**UA-DLLME ^a^-GC-NCI-MS**	Acetone (1000 μL)	5	2	5	/	17.5	1.0–200 (BDEs 28, 47, 99, and 100); 5.0–200 (BDEs 153, 154, and 183); 5.0–500 (BDE 209)	70.6–105.1	[45]
**SFOME ^b^-HPLC-VWD**	/	40	25	/	10	26.3	500–75,000 (BDEs 28, 47, 99, 154, and 183); 5000–500,000 (BDE 209)	92.0–118.0	[47]
**DLLME ^c^-HPLC-VWD**	Acetonitrile (1000 μL)	5	<0.5 (equilibrium 1 h)	5	/	32.0	50–50,000 (BDEs 28 and 99); 100–100,000 (BDEs 47 and 209)	87.0–119.1	[43]
**DLLME ^c^-GC-EI-MS**	Acetonitrile (1000 μL)	25	<0.5	5	/	27.0	5.0–10,000 (BDE 100)	91.0–107.0	[19]
**EA-DLLME-SAP ^d^-GC-MS-MS**	/	5	<0.5	5	10	13.0	1.0–100 (BDEs 28, 47, 99 and 100); 2.0–200 (BDEs 153, 154, and 183); 5.0–500 (BDE 209)	67.2–102.6	This work

^a^ UA-DLLME: Ultrasound-assisted dispersive liquid-liquid microextraction; ^b^ SFOME: Solidification of floating microextraction; ^c^ DLLME: Dispersive liquid-liquid microextraction; ^d^ EA-DLLME-SAP: Effervescent-assisted dispersive liquid-liquid microextraction based on solidification of the aqueous phase.

## Data Availability

The data presented in this study are available in insert article.

## Supplementary Materials

The following are available online. Figure S1: GC-MS-MS chromatogram of blank samples and PBDEs standard mixture solution.

## Abbreviations

PBDEs	Polybrominated diphenyl ethers
EA-DLLME-SAP	Effervescent-assisted dispersive liquid-liquid microextraction with solidification of the aqueous phase
GC-MS-MS	Gas Chromatography-Tandem Mass Spectrometry
GC-MS	Gas Chromatography-Mass Spectrometry
LLE	Liquid-liquid extraction
SPE	Solid-phase extraction
LPME	Liquid phase microextraction
SPME	Solid phase microextraction
SBSE	Stir bar adsorption extraction
DLLME	Dispersive liquid-liquid microextraction
EA-DLLME	Effervescent-assisted dispersive liquid-liquid microextraction
DLLME-SFO	Dispersive liquid-liquid microextraction based on the solidification of floating organic drop
DLLME-SAP	Dispersive liquid-liquid microextraction with solidification of the aqueous phase
LOQ	Limit of quantification
AEI	Advanced Electron Ionization
