# Supplementary material for: Determination of Polybrominated Diphenyl Ethers in Water Samples Using Effervescent-Assisted Dispersive Liquid-Liquid Icroextraction with Solidification of the Aqueous Phase
Source: Molecules. 2021 Mar 4;26(5):1376. doi: 10.3390/molecules26051376 (PMC7961388; doi:10.3390/molecules26051376)
Supplement: Supplementary file 1 [file molecules-26-01376-s001.pdf]

Supplementary material

Determination of polybrominated diphenyl ethers in water samples using effervescent-assisted dispersive liquid-liquid microextraction with solidification of the aqueous phase

Yue Wang [1 2] &, Qicai Zhang [2], Shanshan Chen [2], Lin Cheng [2], Xu Jing [3], Xianli Wang [2], Shuhui Guan [2], Weiguo Song [2,*] and Qinxiong Rao [2,*]

[1] Colllege of Food Sciences, Shanghai Ocean Universidy, Shanghai 201306,China
[2] Institute for Agro-food Standards and Testing Technology, Shanghai Academy of Agricultural Science, Shanghai 201403, China
[3] College of Food Science and Engineering, Shanxi Agricultural University, Taigu, Shanxi 030801, P.R. China
* Correspondence: songweiguo@saas.sh.cn; qinxiongrao@163.com

Number of pages: 2

Number of figures: 1

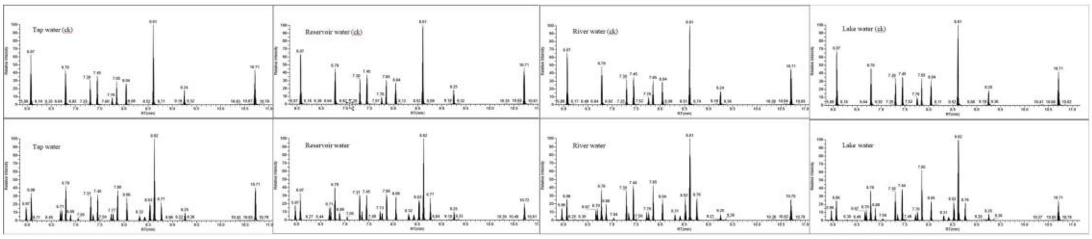

Figure S1. GC-MS-MS chromatogram of blank samples and PBDEs standard mixture solution.
